# Foregrounding Communication Access in Person-Centred Decision Making for People with Motor Neurone Disease: A Narrative Review

**DOI:** 10.3390/healthcare14152307

**Published:** 2026-07-31

**Authors:** Camille Paynter, Susan Mathers, Adam Vogel, Madeline Cruice

**Affiliations:** 1 School of Health Sciences, University of Melbourne, Melbourne, VIC 3010, Australia; 2 Calvary Health Care Bethlehem, Melbourne, VIC 3162, Australia; 3 Redenlab Ltd., Melbourne, VIC 3000, Australia; 4 School of Health & Medical Sciences, St. George’s University of London, London EC1V 0HB, UK

**Keywords:** motor neurone disease, amyotrophic lateral sclerosis, communication impairment, shared decision making, person-centred care, carers, narrative review

## Abstract

**Highlights:**

**What are the main findings?**

- For people living with MND (plwMND) and their family carers, significant effort is required to compensate for communication impairments and prepare for clinical appointments. This effort may go unnoticed by healthcare professionals, as much of it occurs outside of clinical environments.
- When communication is slow or effortful, expression of nuanced preference is reduced. A failure to accommodate communication needs risks inadvertently transfering decision making away from plwMND. Decision making may be facilitated by clinicians explicitly communicating the shared responsibilities of shared decision making.

**What are the implications of the main findings?**

- Strategies are provided for clinicians and for people affected by MND. PlwMND and family carers should be empowered to be explicit about their values, preferences, and how clinical care can be adjusted to meet their needs. This may need to be explicitly explained.
- An ALS/MND multidisciplinary decision making model is presented which foregrounds communication skills to facilitate autonomy and person-centred care.

**Abstract:**

Effective motor neurone disease (MND) management depends on patient and carer involvement in decisions about interventions and future care. Communication and cognitive impairments are common in MND and have under-recognised consequences for shared decision making and autonomy. This narrative conceptual review draws on empirical qualitative research with people living with MND and unpaid family carers and the literature specifically concerning shared decision making and communication in MND. Themes relating to communication, information use, and decision making styles were mapped onto an ALS/MND multidisciplinary decision making model. Enhancements to the model include expanding the decision making context beyond in clinical activity, embedding communication and cognitive skills and accommodations across stages, and acknowledging risks to collaborative decision making. Practical strategies for clinicians, healthcare services, people living with MND, and family carers are proposed to ensure that communication is foregrounded in-person-centred MND care. Observational and implementation research is required to evaluate and refine the proposed approaches.

## 1. Introduction

Motor neurone disease (MND), or amyotrophic lateral sclerosis (ALS), is a progressive neurodegenerative condition with insidious onset and heterogeneous presentations [1]. Degeneration of motor neurones causes progressive arm and leg weakness, speech, swallowing, and respiratory impairment [1]. Historically approached as a pure motor disorder, data now suggests that 25 to 50% of people will be affected by cognitive, linguistic, and/or behavioural changes during the disease course [1,2]. Over 80% of people living with MND (plwMND) will experience communication difficulties [3]. Communication impairment results in significant vulnerability and increases reliance on carers for communication support [4,5]. (In this paper, unpaid family carers will be described as ‘carers’.) Whilst communication impairments may be obvious, the cognitive status of plwMND may not be well recognised by families or clinicians. Underestimating cognitive and behavioural deficits risks poorer healthcare outcomes: MND with frontotemporal dementia shortens survival, and executive dysfunction reduces intervention adherence [6].

With no curative treatment for MND, clinical care emphasises symptom management, assistive technologies, quality of life, and advanced care planning. People living with MND (plwMND) and their carers are required to engage in complex healthcare decisions across the illness trajectory, often making anticipatory choices about interventions such as non-invasive ventilation, gastrostomy, and assistive communication technology [7,8,9]. Collaborative, person-centred care focused on personal values is essential as disease progression pervades quality of life, and impacts carer and family support [5,10,11]. Decision making has been described as cyclical as plwMND adapt and manage ongoing change [12]. Early discussions are encouraged as some interventions are time-sensitive (e.g., gastrostomy) [11] or have improved clinical benefit with early adoption (e.g., assistive communication technology implementation) [13]. Adoption of proactive decision making style is encouraged to prepare for unavoidable change [14].

Clinical guidelines recommend a coordinated team approach for care [11]. Multidisciplinary team clinics have been shown to enhance survival compared with care delivered in general neurology clinics [10,11]. Multidisciplinary specialist clinicians are skilled at addressing the changing needs of patients and enhancing collaboration between patients, families, and clinicians [15]. However, the communication and cognitive impairments commonly associated with MND create distinctive barriers to meaningful participation in healthcare discussions [14,16,17]. Clinical guidelines offer limited practical support for addressing these impairments (e.g., NICE 2019) [18] and some guideline sets do not prioritise communication support. For example, the American Academy of Neurology Institute (AANI), which develops evidence-based quality measures to standardise clinical performance metrics, retired the measures ‘ALS cognitive and behavioural impairment screening’ and ‘ALS communication-support referral’ in its 2022 review [19]. Separately, the empirical literature has not sufficiently articulated how communication and cognitive deficits alter the mechanics of decision making or specified how services should adapt to preserve autonomy [9,20,21]. There is a need to translate lived-experience findings into actionable clinical guidance.

To address the gaps in clinical guidance and the empirical literature, this narrative conceptual review synthesises empirical literature on participation in healthcare decision making in MND focusing on lived-experience, and foregrounds the importance of communication. While we reviewed current clinical guidelines, these documents did not offer detailed, practical guidance on how cognitive and communication impairments specifically affect decision making processes and healthcare involvement in MND or how clinicians could adapt to preserve autonomy. Further, clinical guidelines do not present the lived-experience of plwMND and carers. We provide support strategies for clinicians and people affected by MND that will facilitate person-centred care, optimise healthcare involvement, and accommodate communication needs specific to the MND context. Lastly, we explicitly represent communication access in an existing MND decision making model, recognising that communication supports and barriers directly shape information exchange, expression of preferences, and enactment of healthcare decisions.

## 2. Methods

This manuscript adopts a narrative conceptual review approach. We conducted a structured, purposive search to synthesise empirical literature on the impact of communication and/or cognitive impairment on healthcare involvement and decision making in motor neurone disease (MND). Searches were run in MEDLINE (via OVID), CINAHL, and PsycINFO for peer-reviewed research from 1 January 2006 to 31 March 2026. Search terms used were either as MESH or subject-heading terms: motor neuron* disease OR amyotrophic lateral sclerosis AND decision making AND (communication disorder or communication impairment) OR (cognitive disorder or cognitive impairment). Searches were limited to English language publications due to resource constraints. Eligible studies needed to address issues of communication impairment or cognitive impairment in relation to healthcare involvement and/or decision making from the perspectives of plwMND or carers. We excluded studies purely describing clinical assessment of cognitive function and involvement in clinical trials. Review articles were excluded; however, to ensure comprehensive identification of primary studies we used targeted citation-tracking (using SCOPUS) of topic-relevant reviews to locate additional empirical studies. A PRISMA flow diagram is provided in Supplementary File S1.

Included studies were analysed using inductive content analysis; findings were extracted, thematically or categorically organised, and themes presented. Salient concepts within themes were mapped onto an established MND/ALS decision making model [15] to foreground communication access. Further, we provide practical strategies for clinicians, healthcare services, patients and carers to improve shared decision making understanding and access. Our objective was to generate conceptually coherent, clinically applicable and practical strategies rather than to perform a systematic review. As this review did not involve human subjects, ethical approval was not required.

## 3. Results

Eighty-four articles were screened: 68 articles were excluded following title or abstract review due to being duplications, off-topic, reviews, case studies, opinion papers, assessment of cognition, or clinical trials. Sixteen full-text articles were assessed for eligibility with seven excluded with reason (see Supplementary File S1). Nine papers met our inclusion criteria; eight empirical qualitative studies and one clinical/service paper (see Supplementary File S1 for summary). Some of the studies cited in the Introduction were identified through our searches and are included in the narrative synthesis; all included studies are listed in Supplementary File S1 Table S1.

Included studies were published between 2009 and 2024 and were conducted primarily in Australia (six studies), with additional work from Ireland, England, and the USA. Participants included plwMND, carers, and healthcare professionals; all empirical studies addressed communication and/or cognitive impairment in relation to healthcare involvement and decision making, while the clinical paper contextualises practice implications for decision making in the context of communication and/or cognitive impairment.

Inductive content analysis of the nine included sources produced two principal analytical themes: (1) “Communication shapes MND decisions”, and (2) “Heterogeneous decision making approaches and dynamic information preferences”. These themes highlight the central role of communication, decision making style, and information preferences in shaping involvement-shared decision making in MND for plwMND and carers and how it might influence clinical interactions.

From the thematic synthesis, we generated two applied outputs. First, pragmatic strategies and clinician prompts (presented in Table 1) that operationalize the empirical findings to support communication-accessible, person-centred decision making (Section 3.2). Second, we mapped salient concepts onto an established MND/ALS decision making model (Section 3.3) and identified three areas for enhancement: (a) expanding the decision making context beyond clinic activity, (b) providing specific strategies to navigate barriers to shared decision making, and (c) identifying risks to effective participation.

The following subsections present the analytical themes, followed by the derived strategies, and the proposed model enhancements.

### 3.1. Communication, Decision Making Style, and Information Preferences

The following themes capture factors that influence shared healthcare decision making participation for plwMND, carers, and clinicians.

#### 3.1.1. Communication Shapes MND Decisions

People living with MND and carers regard communication as essential to being heard, directing care, and exercising autonomy [22,23]. Significant effort is required to employ compensatory strategies or preparatory activities for both plwMND and carers although this effort may go unnoticed to healthcare professionals, as much of it occurs outside of clinical environments [17]. The effort to communicate often results in diminished communication for plwMND, which compromised participation in healthcare interactions [17]. Strategies and accommodations are necessary for plwMND to maximise their capacity to independently manage their own healthcare. Without these accommodations, patients risk being excluded as their speech declines which contributes to anticipatory anxiety about future inability to direct care [23,24]. The preparatory and compensatory tasks undertaken outside clinical encounters include drafting phrases and questions, preparing alternative and augmentative communication devices (AAC), coordinating family deliberation, and managing fatigue around appointments [17]. Much of this work may be unseen by clinicians; yet, it is essential for enabling participation. Failure to acknowledge or ameliorate this burden may compound inequity, especially for patients without available carers.

When conversational fluency is lost or AAC output is slow, opportunities for real-time clarification, negotiation, and expression of nuanced preference are reduced. Patients may therefore acquiesce to clinician recommendations rather than actively deliberate [17,23]. Carers frequently support communication by acting as interpreters, summarising options, and advocating [25]. Given the time-sensitive nature of many MND interventions, failure to accommodate communication needs risks missed opportunities and can inadvertently transfer decision control to clinicians or carers rather than the plwMND. Trust and continuity with clinicians shape whether plwMND feel safe to express nuanced preferences, making relational continuity especially important when communication is impaired [26].

#### 3.1.2. Heterogeneous Decision Making Approaches and Dynamic Information Preferences

People living with MND and carers often perceive that there are limited clinical decisions to make because options are constrained by disease progression and lack of cure [23]. This may result in plwMND and carers taking a passive role in shared decision making [27]. Clinicians may therefore need to explicitly communicate the shared responsibilities for effective decision making. Decision making preferences among plwMND and carers are highly individual [23]. Some people adopt a collaborative style, actively seeking and sharing information and deliberating with family between appointments, a pattern more common where participants or relatives have scientific or medical backgrounds. Others prefer to decide independently and may not involve family, even when living with them, and some do not share clinical information with relatives [23,25,28]. These findings caution clinicians against assuming decision making roles from living arrangements or background and underscore the value of eliciting each person’s preferred approach.

People living with MND and carers can differ in the amount, timing, and format of information they want [28]. Some preferred technical high-level information and detail, whereas others sought easy-to-understand information, staged deliberately to allow reflection [28]. Some individuals intentionally avoided information about prognosis or future interventions as an emotional coping mechanism. Limitations of health literacy or accessing less credible sources together with avoidance of information can hinder engagement in healthcare [15,28]. Nuanced information is therefore needed and will depend on the emotional readiness, attitudes, and preferences of plwMND and carers, acknowledging that their individual readiness, attitudes, and preferences may be different.

Decision making in MND is described as a cyclical process in which people perceive and appraise change, select active or passive adaptation strategies, and re-evaluate outcomes as new changes occur [12]. Establishing a preferred decision making style for plwMND and carers, alongside their information preference, is important to foster shared decision making [28]. Clinically, this implies the need for time-sensitive approaches: clinicians should reassess decision making preferences and information readiness at key transition points rather than assuming a stable preference over time [12,28]. Furthermore, clinicians should explicitly highlight time-sensitive intervention options so that plwMND can make informed choices while windows of opportunity remain [23,27]. Achieving this within busy specialist clinics requires deliberate processes to gather and maintain personalised information while preserving continuity of care.

### 3.2. Enhancing Shared Decision Making; Strategies Informed by the Perspectives of plwMND and Carers

The broad healthcare literature and MND clinical guidelines advocate for person-centred care and promote shared decision making. Shared decision making is based on the paradigm of interaction between healthcare professionals and patients, and it is largely assumed this is enacted through verbal communication. In this context, if communication impairment is not acknowledged and accommodated, then the shared decision making process will be compromised.

Information provided to plwMND and carers to facilitate shared decision making needs to be nuanced as *a one size fits all* approach which will not meet individual needs [17,27,28]. Consequently, healthcare professionals (HCPs) need to understand the needs of the person (i.e., both plwMND and carers). Clinicians’ awareness of decision making styles, information preferences, and communication needs will be facilitated by ‘*getting to know*’ the person [17,28].

Data extracted from the included empirical studies were synthesised to generate pragmatic strategies and prompts for clinicians *and* plwMND *and* carers. Building on Kissane’s (2010) evidence-based consultation framework [28,29], these strategies operationalize the review findings and are presented as a support tool in Table 1. The supporting evidence is cited in the main text and Supplementary File S1 rather than within the table itself.

One of the aims of providing results with suggestions for HCPs and people affected by MND is to empower plwMND and carers in clinical appointments which may encourage them to be explicit about their values, preferences, and how clinical care can be adjusted to meet their needs. These supportive strategies facilitate person- and carer-centred care, optimise involvement in healthcare, and accommodate communication needs specific to the MND context.

**Table 1:** Strategies to foster involvement in healthcare and to accommodate communication needs.

Clinical Contact	Healthcare Professional Communication Skills	Patient and Carer Communication Skills
Pre-consultation.	Establish whether patient has any communication needs. Establish patient’s preferred mode of communication; e.g., email, telephone call, SMS/text. Establish patient’s preference for communication outside of clinic with self or family member. Offer option of face-to-face or telehealth appointment. Consider support patient may require to facilitate telehealth appointment. Check if carer/family member attending and identify and resolve barriers if present. Consider setting appointment agenda items prior to appointment; especially important for patients reliant on alternative communication modes. Allocate extra time for consultations and/or consider conducting joint appointments with another HCP for people with communication impairment and/or fatigue.	Advise healthcare professional (HCP) of any communication needs, devices used, and/or helpful strategies. Advise HCP of communication mode preference; e.g., email, telephone call, SMS/text. Advise HCP of main contact for communication, i.e., self or other. Advise HCP if preference for face-to-face or telehealth appointment. If attending multiple appointments at specialist clinic, negotiate appointment intensity preference, e.g., all on one day, spread out over 2–3 days. Consider setting appointment agenda prior to appointment to prepare (especially important if using non-verbal means of communication and/or experiencing significant fatigue).
Pre-meeting checklist for those attending the consultation.	Set agenda items in collaboration with patient/carer/family member. Consider the agenda items may differ between parties. Check preference for information complexity (i.e., scientific or lay terms). Check preference for depth of information, i.e., overview or detail appreciating that this may different between patient and carer/family member. Check communication needs of patient and support required.	What are your three (3) main concerns to discuss at the meeting. Determine who will attend the meeting with you. What communication needs, devices, or support do you have? Advise HCPs so they can prepare in advance. Consider how you like to make decisions (i.e., on your own, collaborate with family, a bit of both, it depends on the decision, you prefer to do what the doctor recommends etc). All options are right if they’re right for you; just explain to your HCP your preference. Can your partner/family member attend? If not, negotiate with HCP how information will be relayed to them.
Start the consultation with setting expectations for the appointment.	Confirm agenda. Check/re-check patient/family preference for decision making involvement and style. Decision making style of patient may be independent of family or collaborative. Consider also this may be a new concept for some patients when discussing. Check preference for information complexity (i.e., scientific or lay terms). Check preference for depth of information (i.e., overview or detail appreciating that this may be different between patient and carer/family member). Explain, non-judgementally, that shared decision making involves collaboration between HCP, patient/carer/family member. Explain their engagement and opinions are an important contribution for shared decision making. Explicitly endorse the asking of questions.	Confirm agenda. Remind HCP of how you prefer to be communicated with. Would you like adjustments? What works best for you? Accommodating for changes in communication and/or thinking skills is important for HCP to understand, explain what you would like to be done differently. Remind HCP of any preferences for using communication devices (AAC), writing, paraphrasing, e.g., *do/don’t anticipate my words if I’m struggling, do/don’t read over my shoulder if using AAC*, etc.
During consultation establish understanding of disease status, interventions under consideration, and psychosocial needs and concerns.	Provide information in a sympathetic and empathic way. Check patient and carer/family understanding. Check that they are ready to continue the conversation and/or receive more information. Provide a clear link to why interventions are recommended and how it will help. Provide pros and cons. Ensure patient/carer/family member have equal opportunity (note that patient/carer/family member may have different needs and preferences). Check patient/carer/family understanding individually. Offer more time, decision delay, or abort if patient/carer/family not ready.	Ask questions and express concerns. Ask for clarification if you are unsure of information. Ask for time to process information to reflect on options and information if needed. Remember that decisions are a shared process and can be made according to your style (i.e., on your own, collaborating with family/HCP, a bit of both, it depends etc).
Consider patient, carer/family values, dynamics, and lifestyle factors.	Consider using qualifiers or ‘soft’ suggestions. For example, *“Have you thought about …?”, or “In the future you might need …”, “If [this] happens, you might need…”* Consider incorporating terminology used by patient/carer/family (e.g., the term *‘decision making’* does not resonate with all patients/carers. Acknowledge concern and apprehension as appropriate. Reinforce value of shared decision making.	Discuss family dynamics with HCP. Provide explicit information about how you and your family generally approach ‘big’ or difficult decisions. Discuss your values, lifestyle and what’s important to you. Consider and discuss how these will influence your approach to managing your MND.
Closing and post-consultation.	Summarise information, discuss and confirm next steps. Send written summary of plan/discussion to patient and nominated others. Advocate for patient’s communication preferences and/or limitations with other health services (e.g., include communication preferences in handover reports and referrals).	Summarise information, discuss and confirm next steps. Request written summary of issues discussed and/or plans made. If appropriate state who this should be shared with.

### 3.3. Decision Making Models and Guidelines in MND: Enhancing an MND Person-Centred Decision Making Model

The narrative analysis identified a patient-centred decision making model, the “model of patient-centred decision making in specialised ALS multidisciplinary care” [15] (p. 1778). This model incorporates patients’ cyclic decision making patterns within multidisciplinary settings [12] and facilitates the inclusion of carers [25] across four stages of the decision making process; Participant Engagement, Option Information, Option Deliberation, and Decision Implementation. Whilst Hogden and colleagues capture the complexity of MND decision making in their decision making model, the model does not explicitly recognise the communication skills required for collaborative decision making or the issue of communication impairment for this patient group.

Communication skills need to be foregrounded in decision making models to facilitate autonomy and person-centred care, and because of the high value plwMND and carers place on communication [22,23]. Additionally, communication skills need to be present in decision making models to highlight to clinicians the essential support and accommodations required by some plwMND to maximise access to healthcare [17,23]. Empirical data identified for this review expose three areas for enhancement to Hogden and colleagues’ model of patient-centred decision making in specialised multidisciplinary care which are:

(1) expand the context of decision making to include out-of-clinic decision activity;
(2) provide strategies to navigate barriers to shared decision making; and
(3) identify additional risks to effective shared decision making.

**Figure 1:**
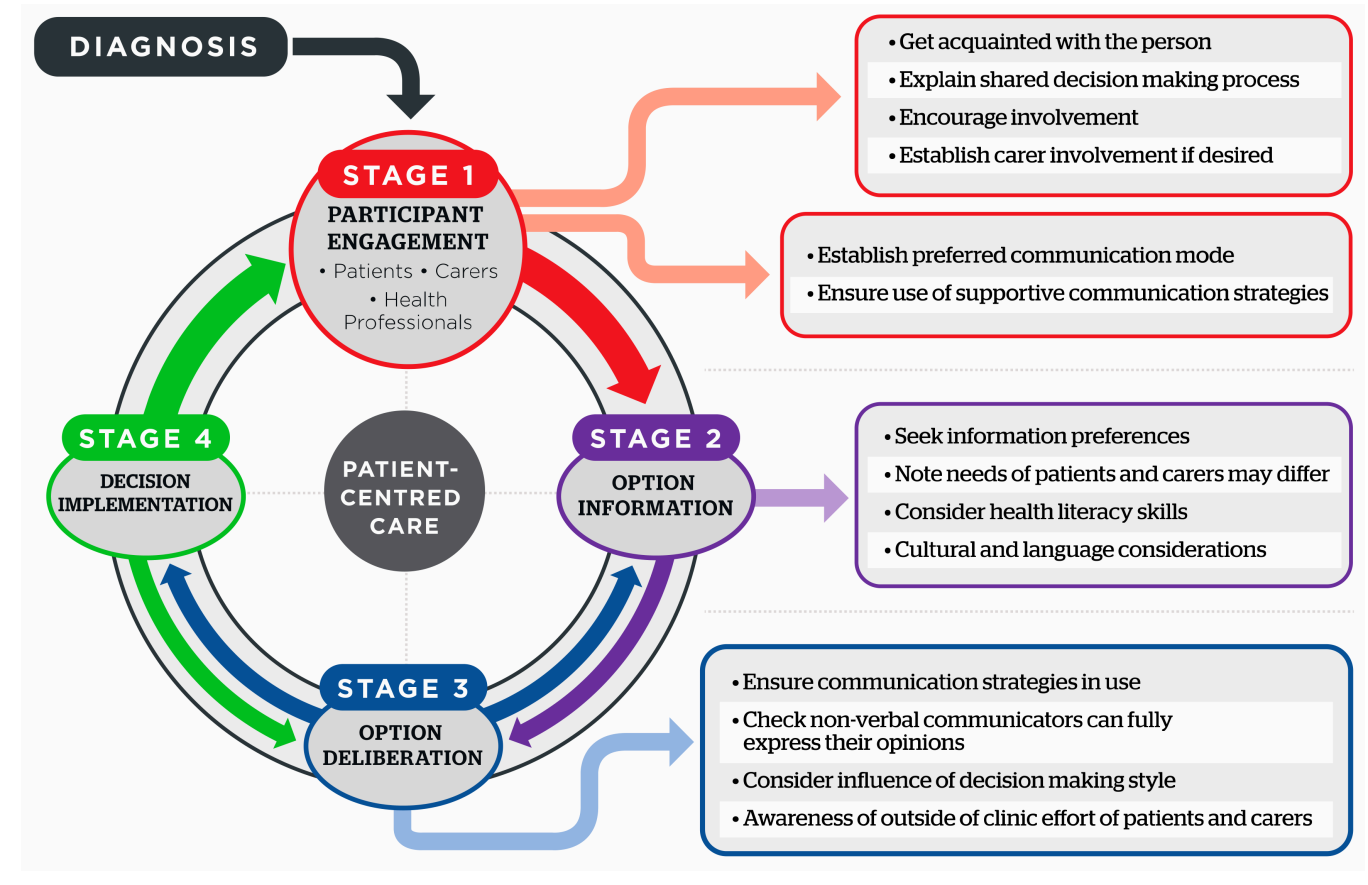
Enhanced model of person-centred decision making in MND/ALS.

These suggestions (explained below) extend the existing version of the model and result in an enhanced model of person-centred decision making in MND/ALS (Figure 1).

#### 3.3.1. Expand Context of Decision Making

Healthcare decision making is often viewed as being situated *within a multidisciplinary setting* of a specialised clinic. However, decision making in MND is not confined to clinic walls; plwMND and carers report that considerable activity is undertaken *outside of the clinic environment* to participate in healthcare. Patients and carers engage with healthcare information, deliberate about decisions, and undertake preparation to facilitate involvement in-clinic-based appointments independent of clinic appointments.

Preparatory work (e.g., agenda setting, AAC device preparation), asynchronous information exchange and family deliberation occur in community contexts and shape in-clinic participation. The enhanced model explicitly references some of this ‘out-of-clinic’ preparation. HCPs may wish to consider the use of: appropriate need assessment tools; digital technology and telehealth to engage other family members and/or other HCPs unable to attend in-person consultations; pre-clinic checklists (such as provided in Table 1); and pre-clinic triage contact (via email or telephone) to address expectations, set agenda and attendance, establish communication/other needs, and carer concerns.

#### 3.3.2. Provide Strategies to Navigate Shared Decision Making

Rather than treating communication or cognitive issues as a peripheral issue, the enhanced model foregrounds systematic assessment and accommodation of communication and cognition across participant engagement, option information, and option deliberation. Suggested strategies are provided specifically for Stages 1–3 of the model (note that suggestions are not provided for Stage 4 ‘Decision Implementation’ as it is assumed that decision making is being enacted once prior stages are addressed).

Suggested strategies to enhance Participant Engagement (Stage 1):

- Get acquainted with the person (the patient, carer, and family member) with acknowledgment that their needs and preferences may be different.
- Establish preferred mode of communication. Adjust usual practice, and provide support required to address and/or minimise the impact of comm/cog impairment.
- Ensure supportive communication strategies are in place for patients reliant on alternative communication modes to enable patients to fully express their opinions.
- Explicitly explain the shared decision making process and encourage active involvement.
- Identify any barriers to carer involvement and offer remediation where possible.
- Consider conducting consultations in a different way. Recognise the potential value telehealth consultations can offer to facilitate the involvement of others (e.g., family members residing interstate or abroad, and/or local or interdisciplinary HCPs) who wouldn’t typically attend an in-person consultation.

Suggested strategies to enhance Information Provision (Stage 2):

- Seek patients’ and carers’ information preferences. Consider complexity, timing and relevance of information provided, noting that needs and preferences of plwMND and carers may be different.
- Consider health literacy skills of patient and carer, noting that health literacy is content- and context-specific [30] and therefore may change depending on option.
- Consider the needs of people from culturally and linguistically diverse (CALD) backgrounds.

Suggested strategies to enhance Intervention Deliberation (Stage 3):

- Ensure patients reliant on non-verbal communication modes can fully express their opinions (i.e., supportive communication strategies are in place).
- Acknowledge support provided by carers to enable deliberation and offer assistance and support.
- Be aware of the ‘invisible’ activity that plwMND and carers undertake outside of clinic appointments.
- Consider influence of decision making style (e.g., independent, collaborative, needs support, mixed).
- Consider health literacy skills of patient and carer, noting that health literacy is content- and context-specific [30] and therefore may change.

#### 3.3.3. Identify and Acknowledge Risks to Effective Participation

Effective shared decision making depends on recognising and explicitly acknowledging factors that may impede participation at each stage of the decision making process. Key risks for Stages 1–3 are considered below. While targeted strategies can mitigate many of these hazards, acknowledging them early is essential to tailor supports and safeguard the integrity of collaborative decision making.

Potential risks identified for Participate Engagement (Stage 1) include poor dynamics between participants (i.e., patient, HCP, carer/family member), insufficient support to accommodate communication impairment, inability to engage with digital communication technology and telehealth, and unavailable or there are no family member/s to provide necessary assistance [17,28]. Whilst the above suggestions will not eliminate the risk of poor dynamics, some strategies may ameliorate issues if they do occur.

Risks identified for Option Information (Stage 2) include changes in a patient’s cognitive skills, and plwMND and carers placing confidence in information sources with questionable credibility [15]. Further risks include poor health literacy and information avoidance [28]. Moreover, the provision of information not sufficiently nuanced to needs (e.g., to the point that plwMND or carers feel overwhelmed) may lead to a breakdown of this process and could be considered another risk to the success of Stage 2.

Communication impairment, cognitive and behavioural change, and prolonged deliberation have been identified as risks to Option Deliberation (Stage 3) [15,17,23]. People living with MND who are reliant on non-verbal means of communication do not always express their opinions in full or have the opportunity to express them accurately. The strategies provided to enhance this stage of the decision making model (i.e., accommodating needs, identifying decision making style and acknowledging support) may in fact facilitate deliberation and reduce the risk of deliberation becoming prolonged.

## 4. Generalisability and Application in Diverse Clinical Settings

While the strategies and decision making model enhancements presented here were derived from studies conducted predominantly in specialist multidisciplinary MND services, their underlying principles are intended to be adaptable across a range of healthcare settings and cultural contexts. Central to this is the principle that communication and cognitive support are shared responsibilities of all clinicians (e.g., “getting to know” the person). This framing discourages the diffusion of responsibility that can occur when teams assume communication support is the remit of a single discipline. We also emphasise the importance of empowering plwMND and carers to participate actively in shared decision making. This goal may require explicit explanation and support to build confidence, and clarification of decision making roles.

Nevertheless, implementation will require local adaptation. In resource-limited settings or where specialist MND clinics are not available, teams may consider adopting pragmatic, low-cost approaches to protect communication access and autonomy such as those suggested in Table 1.

## 5. Conclusions

Communication and cognition are central determinants of meaningful involvement in MND healthcare decisions and need to be foregrounded in clinical care and procedures. Current practice frequently under-recognises the impacts of communication impairment and the considerable invisible labour performed by patients and carers. Enhancing person-centred decision making models to consider out-of-clinic activity undertaken by plwMND and carers, embed accommodations across decision making stages, and to consider that additional risks to collaboration provide a practical guide for HCPs and healthcare services. Implementing simple administrative and communication changes offers immediate potential to improve participation and protect autonomy. Making considerations to accommodate communication and cognitive change requires modest structural and cultural shifts but can be embedded into routine practice to enhance shared decision making especially for plwMND presenting with communication impairments.

## 6. Limitations

Narrative reviews allow flexibility to explore conceptual developments which increases the risk of selection and reporting bias and can make findings less reproducible. Recommendations and strategies are presented as pragmatic proposals to be evaluated empirically. Our recommendations derive mainly from qualitative studies conducted in specialist settings; their transferability to other healthcare systems and low-resource contexts should be established through local adaptation and empirical evaluation.

## 7. Research Priorities

Future research exploring how HCPs *actually* support plwMND who have communication or cognitive impairment is needed and would be well served using ethnographic methodology, where actual interactions are observed. Findings from such studies could directly inform the development of targeted training and resources for HCPs. An ethnographic approach would facilitate inclusion of plwMND with cognitive deficits and participants from culturally and linguistically diverse backgrounds. Separately, co-design and feasibility methods could be employed to actively engage these stakeholders in identifying priorities and designing and implementing improvements to their involvement in healthcare. Ideally this implementation research would occur in both specialist and non-specialist clinics, and include resource-constrained and culturally diverse settings.

## Data Availability

This manuscript is a narrative review and did not generate new primary data. All sources are cited in the reference list; search PRISMA flow diagram is provided in Supplementary File S1.

## Supplementary Materials

The following supporting information can be downloaded at https://www.mdpi.com/article/10.3390/healthcare14152307/s1, Table S1: Source data; Figure S1: PRISMA flow diagram summarising the literature search.

## Abbreviations

MND, Motor Neurone Disease; AAC, Augmentative and Alternative Communication; plwMND, People living with MND; Carers, Unpaid family carers. A note on terminology used in this manuscript: motor neurone disease (MND) is the name given to a group of neurodegenerative diseases of which amyotrophic lateral sclerosis (ALS) is the most common. Globally, the terms MND and ALS are often used interchangeably. In this manuscript, the author uses the term MND unless referring to research specific to people living with ALS or authors who have used the term ALS.
