# Supplementary material for: Foregrounding Communication Access in Person-Centred Decision Making for People with Motor Neurone Disease: A Narrative Review
Source: Healthcare (Basel). 2026 Jul 31;14(15):2307. doi: 10.3390/healthcare14152307 (PMC13464704; doi:10.3390/healthcare14152307)
Supplement: Supplementary file 1 [file healthcare-14-02307-s001.zip › supplementary.pdf]

Foregrounding communication access in person-centred decision making for people with motor neurone disease: a narrative review.

Supplementary File S1: **Table S1: Source data**

First Author	Year of publication	Method	Country of research	Participants in research
King	2009	Qualitative interviews	Australia	PlwMND N=25
Hogden	2013	Qualitative interviews	Australia	Family carers of plwMND n=8
Hogden	2015	Qualitative interviews	Australia	PlwMND n=8, family carers n=14, HCP n=32
Judge	2018	Qualitative focus groups	England	PlwMND n=6 and family carers n=7
Lisiecka	2021	Qualitative interviews; multiple interviews (n=44)	Ireland	PlwMND n=10
Paynter [23]	2022	Qualitative interviews	Australia	PlwMND n=19 and family carers n=15
Paynter [17]	2022	Qualitative interviews; longitudinal study	Australia	PlwMND n=19 and family carers n=15
Paynter [28]	2022	Qualitative interviews Longitudinal study	Australia	PlwMND n=19 and family carers n=15
Donohue	2024	Clinical focus	USA	n/a

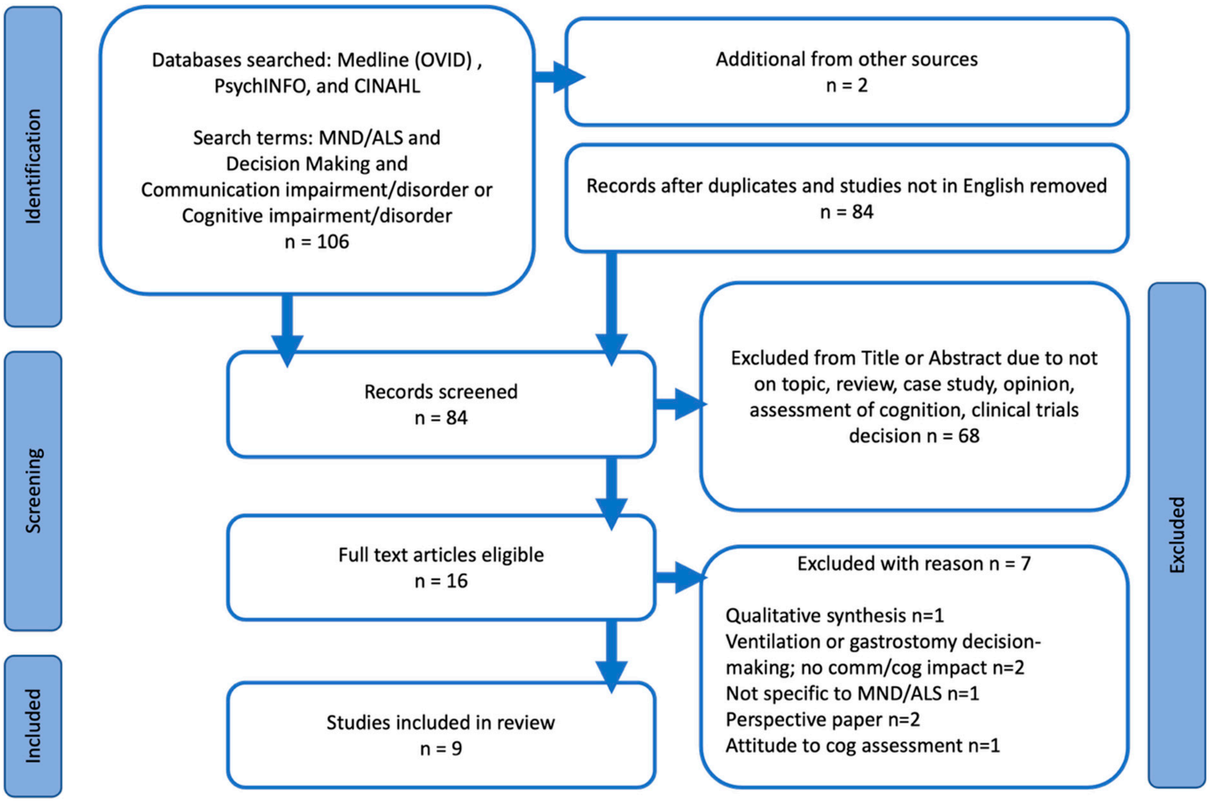

Supplementary File S1 - **Figure S1: PRISMA flow diagram summarising the literature search**
